# A rare presentation of urogenital tuberculosis leading to obstructive uropathy and renal failure: a case report

**DOI:** 10.1186/s12894-025-01876-7

**Published:** 2025-10-27

**Authors:** Abdul Qadir, Nagham Sadik, Mohammad Ghabashneh, Husien Almarawi, Mamunul Islam

**Affiliations:** 1 Department of Medicine, Hamad Medical Corporation, HGH, Doha, P.O. Box 3050 Qatar; 2 Medical Education Department, Hamad Medical Corporation, Doha, Qatar

**Keywords:** Urogenital tuberculosis (UGTB), Extrapulmonary tuberculosis (EPTB), Obstructive uropathy

## Abstract

**Background:**

Urogenital tuberculosis (UGTB) is a rare form of extrapulmonary tuberculosis (EPTB) affecting the kidneys, ureters, and bladder. Its nonspecific symptoms often mimic common urological conditions like urinary tract infections (UTIs), leading to delayed diagnosis. This case highlights the diagnostic challenges and emphasizes the need for timely intervention to prevent irreversible renal damage.

**Case presentation:**

A 43-year-old south-Asian female presented with sudden decreased consciousness and severe renal impairment, requiring emergency dialysis. Despite the absence of typical tuberculosis symptoms or risk factors, imaging revealed obstructive uropathy with hydronephrosis. Urine polymerase chain reaction (PCR) confirmed UGTB. Early interventions, including percutaneous nephrostomy, J-stent placement, and anti-tuberculosis therapy (ATT), resulted in significant clinical improvement.

**Conclusions:**

UGTB should be considered in patients with unexplained urological conditions, particularly in tuberculosis-endemic regions. Early diagnosis and management are crucial to avoid irreversible complications and improve outcomes.

## Introduction

Tuberculosis (TB), caused by Mycobacterium Tuberculosis, is preventable and curable disease. However, it is also second most leading cause of death by a single infectious agent after Coronavirus disease [1]. Amongst the total population, a quarter is estimated to be infected [2]. TB typically infects the lungs (pulmonary TB) but can also infect other sites, extrapulmonary tuberculosis (EPTB).

Urogenital tuberculosis (UGTB) is a rare form of EPTB, accounting for 15–20% of cases, primarily affecting the kidneys, ureters, and bladder [3, 4]. It typically affects the kidneys first, followed by downward spread to the ureters, bladder, prostate, epididymis, or genital tract. Infections may remain clinically silent for years due to the slow progression of renal and urological damage, often leading to diagnostic delays (5,6,). Nevertheless, diagnosis is often challenging due to its nonspecific symptoms—such as dysuria and hematuria—that mimic more common urological conditions like urinary tract infections [4].

TB involving the urologic system has two forms, the most common presentation involves the urinary collecting system (renal pelvis, calyces, ureters and bladder) and the less common one involves kidney lesions (ulcers, abscess formation, perinephric spread, caseating necrosis and calcification) [5]. The onset of UGTB is insidious, the average time period from pulmonary infection and clinical manifestations of UGTB is 22 years [6] with a male to female ratio 2:1.

Patients without traditional risk factors of TB, physicians often face the challenge of delayed recognition, as they have to go through advanced diagnostic techniques including polymerase chain reaction (PCR) and imaging to confirm the diagnosis [7].

Early diagnosis and initiation of anti-tuberculous therapy (ATT) with full compliance are critical as it ensures favorable outcomes [8]. This case underscores the importance of considering UGTB in the differential diagnosis for patients with atypical presentation and unexplained urological symptoms.

### Case Presentation

A 43-year-old south-Asian female presented to the emergency department with a reduced level of consciousness, as reported by her roommates. According to them, she had been well throughout the day but suddenly became unresponsive and non-communicative around midnight without any jerky movements or episodes of incontinence.

Upon arrival, the patient’s Glasgow Coma Scale (GCS) score was 10 and vitally stable. Blood tests revealed severely abnormal renal function, with a markedly elevated creatinine level (~ 2000 µmol/L), and elevated inflammatory markers (Table 1). Emergency dialysis was initiated via a central venous line, after which the patient regained consciousness. She denied any significant past medical history and had no fever, cough or abdominal pain. She reported frequent vomiting and urination earlier that day, which were later attributed to underlying obstructive uropathy and worsening renal function. She had no history of sick contacts and had returned from Indonesia approximately 8 months prior.

**Table 1:** Laboratory investigations

Parameters	Results	Reference range
WBC	6.3	4.0–10.0 × 10^3/uL
RBC	2.4	4.5–5.5 × 10^6/uL
Hgb	6.2	13.0–17.0 gm/dL
Hct	20	40.0–50.0%
MCV	84.4	83.0–101.0 fL
MCH	26.2	27.0–37.0 pg
MCHC	31	31.5–34.5 gm/dL
RDW-CV	17.1	11.6–14.0%
Platelets	281	150–450 × 10^3/uL
MPV	9.0	9.6–12 fL
Absolute Neutrophil count Auto # (ANC)	5.3	2.0–7.0 × 10^3/uL
Urea	39.7	2.5–7.8 mmol/L
Creatinine	1,937	62–106 umol/L
Sodium	138	133–146 mmol/L
Potassium	8.4	3.5–5.3 mmol/L
Chloride	107	95–108 mmol/L
Bicarbonate	7	22–29 mmol/L
Calcium	1.70	1.6–3.24 mmol/L
Bilirubin T	5	0–21 umol/L
Bilirubin D	< 2	0–5 umol/L
Total Protein	67	60–80 gm/L
Albumin	24	35–50 gm/L
ALK phosphate	38	40–129 U/L
ALT	9	0–41 U/L
AST	7	0–40 U/L
Myoglobin	561	25–58 ng/ml
CRP	9.2	0–5 mg/L
Lactic Acid	0.6	0.5–2.2 mmol/L

*WBC* White Blood Cell, *RBC* Red Blood Cell, *Hgb* Hemoglobin, *Hct* Hematocrit, *MCV* Mean Corpuscular Volume, *MCH* Mean Corpuscular Hemoglobin, *MCHC* Mean Corpuscular Hemoglobin Concentration, *RDW-CV* Red Cell Distribution Width-Coefficient of Variation, *MPV* Mean Platelet volume, *ANC* Absolute Neutrophil Count, *ALK phosphate* Alkaline Phosphatase, *ALT* Alanine Aminotransferase, *AST* Aspartate Aminotransferase, *CRP* C-Reactive Protein

**Fig. 1:**
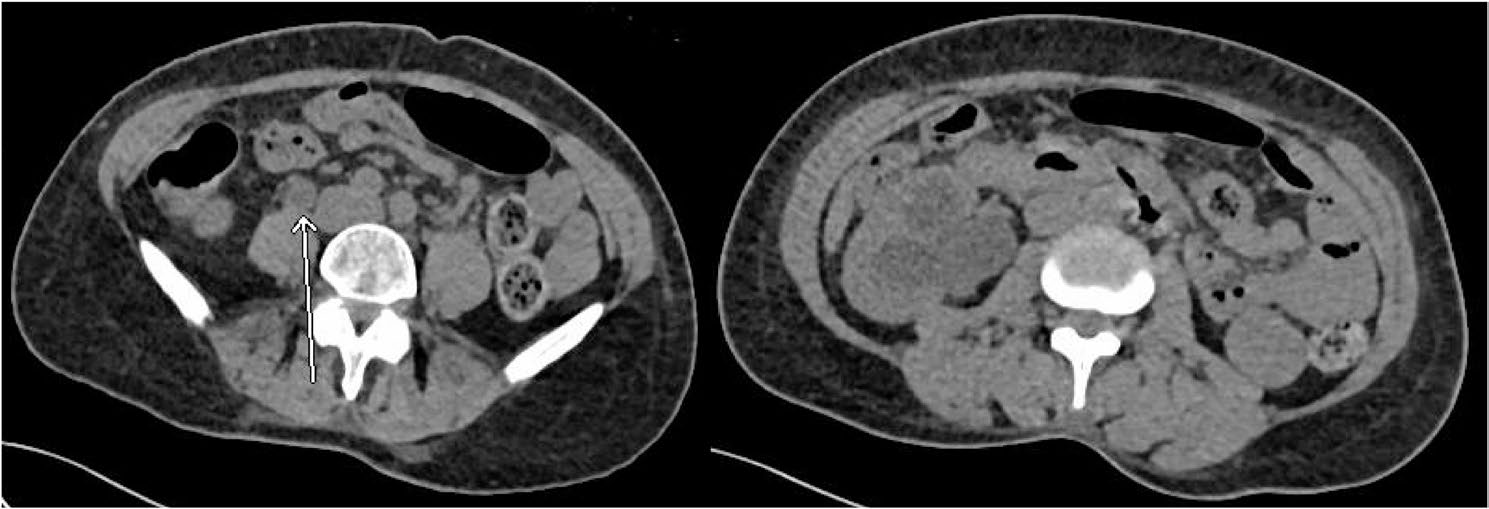
CT scan showing obstructive uropathy with hydronephrosis

**Fig. 2:**
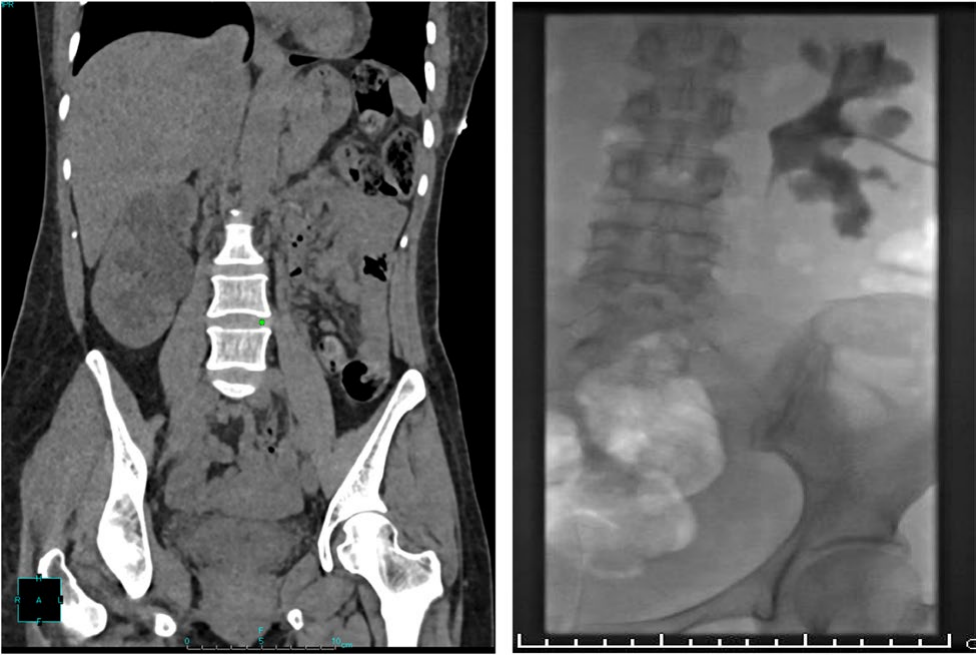
CT scan showing dilated right kidney with hydroureteronephrosis

**Fig. 3:**
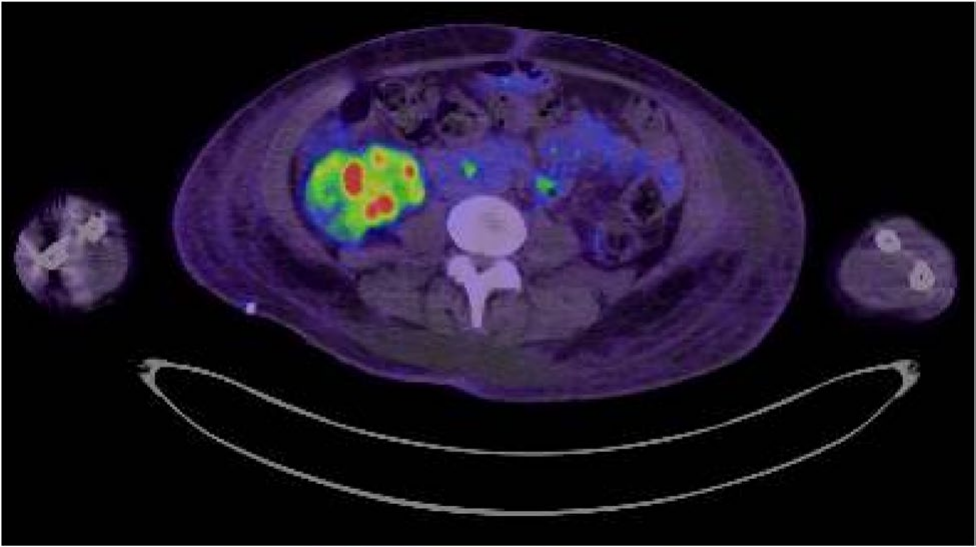
PET scan demonstrates increased metabolic activity in the right renal, consistent with pyelonephritis

A computed tomography (CT) scan of the head was performed to rule out a stroke. Additionally, a CT scan of the urinary tract revealed extensive right hydroureteronephrosis with cortical thinning and scarring, predominantly affecting the upper pole of the kidney, suggesting a chronic obstruction. There was prominent thickening of bladder walls (Fig. 1). To relieve the obstruction, a right-sided percutaneous nephrostomy (PCN) tube was placed. Due to concerns about contrast-induced nephropathy, a contrast-enhanced CT of the abdomen and pelvis could not be performed. The magnetic resonance imaging (MRI) of the abdomen and pelvis without contrast was performed which revealed right nephrostomy placement with a small right perinephric hematoma, diffuse wall thickening of the right renal pelvicalyces and ureter suggestive of pyelonephritis. The left kidney showed a tiny simple cyst with no evidence of hydronephrosis or focal lesions. The ureteral narrowing was most prominent in the proximal to mid-right ureter, with contiguous inflammatory thickening extending from the renal pelvis downward (Fig. 2). As the MRI findings remained nonspecific and could not definitively exclude other causes such as malignancy or atypical infections, a positron emission tomography (PET) scan was performed to further characterize renal involvement and rule out occult malignancy or systemic disease involvement. PET scan demonstrated increased metabolic activity in the right renal parenchyma and perirenal stranding, consistent with pyelonephritis. PET scan was negative for any malignancy (Fig. 3).

Differential diagnoses considered include pyelonephritis, disseminated tuberculosis (TB), urogenital strictures, chronic kidney disease, and autoimmune diseases. Blood cultures and urine cultures were negative. However, urine PCR for acid-fast bacilli was positive, confirming the diagnosis of urogenital TB. PCR was chosen for its high sensitivity and specificity in detecting Mycobacterium tuberculosis in cases with nonspecific clinical and imaging findings, especially when conventional cultures are negative. A double J stent was inserted to relieve the narrowing of the ureteral stricture and to facilitate drainage of the right kidney. The stent was placed successfully without the need for ureteral dilatation or surgical intervention, as the stricture was traversable with guidewire assistance. The patient was diagnosed as UGTB with advanced CKD. She was initiated on anti-tuberculosis therapy (ATT) for 6 months with follow-up urine PCR. She has since shown significant improvement and continues to do well under treatment.

## Discussion

This case report highlights several important diagnostic and management considerations for the clinically heterogenous syndrome that is UGTB. Our patient, who’s already been suffering multiple mild sequelae of undiagnosed UGTB prior to her presentation, was brought to medical attention because of reduced level of consciousness, leading to the diagnosis of very distinct extrapulmonary manifestation of tuberculosis.

An understanding of the pathogenesis is essential for comprehension of range of clinical manifestations of UGTB. Initially UGTB is not associated with specific symptoms; pyuria or hematuria maybe detected as incidental finding. After the disease has progressed to involve the bladder, symptoms of dysuria, urgency and nocturia occurs in half the cases and gross hematuria, low back pain occurs in one-third of the cases [6, 9–11]. Long standing parenchymal involvement may result in tubular proteinuria, which is in subnephrotic range. Systemic symptoms like fever and weight loss are rare. Manifestation of advanced disease is end stage renal disease (ESRD) [11]. In our patient, the likely insidious progression of UGTB led to chronic inflammation, resulting in ureteral narrowing and obstructive uropathy, which progressed to CKD. This asymptomatic presentation with advanced obstructive damage is atypical for UGTB and highlights its diagnostic difficulty. In most reported cases [6, 9–11], patients present earlier with dysuria, frequency, or systemic features. The absence of these signs in our case delayed clinical suspicion until the disease reached a critical stage.

The diagnosis of UGTB should be suspected in patients with relevant clinical manifestations (urinary frequency, dysuria, hematuria and/or sterile pyuria) and relevant epidemiologic factors (history of TB, possible exposure, residence or travel to TB endemic areas) [10]. But our patient had neither of the reported symptoms or etiology that could lead to its definitive diagnosis. A series of radiological imaging and PCR of bodily fluids are essential to rule out broader differential diagnosis [12]. Therefore, we collected the urine samples from our patient for Acid fast bacilli- PCR (AFB-PCR), to establish the diagnosis of UGTB, because AFB-PCR has sensitivity of 87% and specificity of 93–98% out of 100 [13] to find mycobacterium tuberculosis.

TB causes urinary tract inflammation which may lead to obstruction of the collecting system, bladder contraction and development of renal function impairment [3]. There is not much literature for similar clinical presentation of disseminated TB with lower genitourinary tract obstruction, complicated with chronic renal failure while being asymptomatic earlier.

Early stenting and percutaneous nephrostomy is required for patients with ureteral stricture, hydronephrosis and worsening renal functions. While percutaneous nephrostomy and stenting are effective for relieving obstruction, they may be associated with complications such as infection, dislodgement, or bleeding. In chronic cases, reconstructive options such as ureteral reimplantation or nephrectomy may be considered, particularly when renal salvage is not feasible. Anti-tuberculous therapy should also be initiated; TB treatment regimen includes intensive phase of 2 months and continuous phase of 4 months, with isoniazid, rifampin, pyrazinamide, and ethambutol. In our patient, definitive management of the ureteral stenosis involved conservative decompression via double J stenting along with ATT. Surgical correction was not pursued; as the obstruction responded well to this approach both clinically and radiologically.

## Conclusions

In conclusion, this case highlights the importance of considering urogenital tuberculosis (UGTB) in the differential diagnosis of patients presenting with atypical urological symptoms and unexplained renal dysfunction, even in the absence of classical TB risk factors or symptoms.The case demonstrates that early detection, appropriate imaging, and polymerase chain reaction (PCR) testing are crucial for timely diagnosis. This case further emphasizes the need for heightened awareness and clinical vigilance in managing patients with atypical manifestations of extrapulmonary TB, as early diagnosis and treatment can prevent irreversible renal damage.

## Data Availability

The datasets used and/or analysed during the current study are available from the corresponding author on reasonable request.
